# Editorial: New perspectives on osteoclasts in health and disease

**DOI:** 10.3389/fcell.2022.1093394

**Published:** 2022-11-25

**Authors:** Maria-Bernadette Madel, Enrico Iaccino, Claudine Blin-Wakkach, Stefania Mariggiò

**Affiliations:** ^1^ Department of Orthopedic Surgery, Baylor College of Medicine, Houston, TX, United States; ^2^ Department of Experimental and Clinical Medicine, University Magna Graecia of Catanzaro, Catanzaro, Italy; ^3^ Université Côte d’Azur, CNRS, LP2M, Nice, France; ^4^ Department of Biomedical Sciences, Institute of Biochemistry and Cell Biology, CNR, Naples, Italy

**Keywords:** osteoclast differentiation, multinucleated cells, bone resorption, bone signaling pathways, bone tumors, osteoimmunology

## Introduction

Since their discovery 150 years ago, osteoclasts have been the subject of an increasing number of studies. Osteoclasts are indeed very particular because they are among the very few physiologically multinucleated cells, they are the only ones capable of resorbing bone matrix and, although they play a key role in bone remodeling, they belong to the monocyte lineage. Their functions extend far beyond bone resorption, and they are involved in many biological and pathological processes, including osteoporosis, bone tumor and metastasis, rare diseases, and chronic inflammatory diseases. As a result, they remain the subject of intense research, and the number of publications on osteoclasts continues to grow exponentially.

**FIGURE 1:**
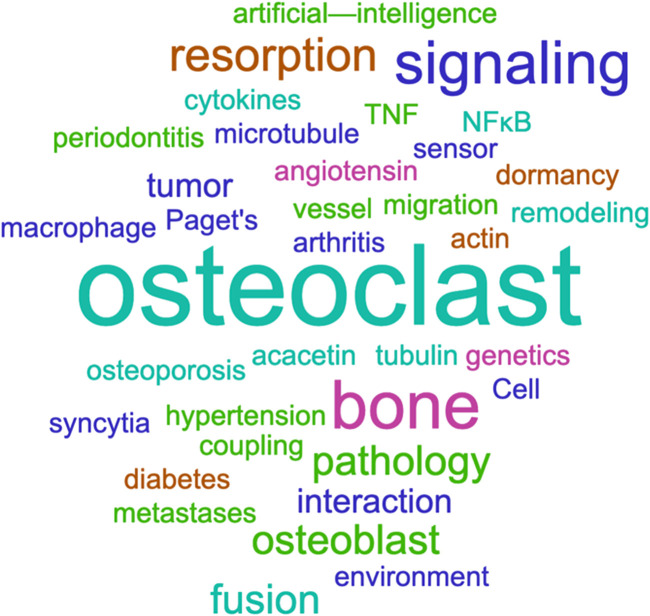
Areas covered by the Research Topic “*New perspectives on osteoclasts in health and disease*”. The word cloud is based on the main keywords and topics of the 13 papers included in this Research Topic. It was built using Word It Out (https://worditout.com/). The word size is proportional to the weight of the word. Colors are random.

The 13 articles in this Research Topic, including 8 Original Research and 1 Method papers plus 3 Reviews and 1 Minireview, to which 97 authors and 28 reviewers from all over the world contributed, reflect the continuing high level of interest in osteoclasts. They bring new data or review recent advances in the regulation of osteoclast differentiation and function, their role in the pathological context and their interest as therapeutic targets, as well as new tools for measuring osteoclast activity and number in *in-vitro* assays (Figure 1).

## Overview

### Cell signaling and molecular regulation of osteoclast differentiation, fusion, and function

At the molecular and cellular level, this topic has contributed to refine membrane receptors and cell signaling in osteoclast physiology and dysfunction, and addressed molecular aspects of osteoclast differentiation, fusion, and function. Bae et al. provided new insights into mTORC1-mediated regulation of osteoclastogenesis and bone resorption, revealing the interplay between MYC and mTORC1 signaling pathways in the regulation of osteoclast resorption. Furthermore, they demonstrated for the first time that the MYC-GADD34 axis is an upstream regulator of mTORC1 activation in osteoclast differentiation, suggesting that this axis is a potential therapeutic target for mTORC1 regulation and pathological bone resorption.

Osteoclast fusion is a tightly regulated process involving cell adhesion and actin reorganization (Dufrancais et al., 2021). Ahmadzadeh et al. summarized recent insights into osteoclast biogenesis, focusing on syncytial cell formation. Osteoclasts are considered the physiological counterpart of the less well-characterized Langhans and foreign body giant cells (Anderson, 2000), formed upon macrophage fusion under pathological conditions (e.g., granuloma or foreign material exposure). Osteoclasts retain several immunomodulatory functions of macrophages, such as phagocytic activity to resorb bone. These observations, together with the osteoclast contribution to bone promotion and angiogenesis, further crushes the image of osteoclasts as specialized cells dedicated exclusively to bone resorption. Two original research articles further explored the mechanisms of cell fusion in osteoclastogenesis. Mangini et al. demonstrated evidence for multimodal regulation of osteoclast differentiation and activity by group IIA secreted phospholipase A_2_ (sPLA_2_-IIA). Using different approaches (KO mice, siRNAs, recombinant proteins, and inhibitors), they reported the involvement of sPLA_2_-IIA in the control of osteoclast differentiation, syncytium formation, and resorption activity in a both catalytically dependent and independent manner. Maurin et al. shed new light on the control of microtubule dynamics in osteoclasts using an osteoclast KO model for TUBB6, a β-tubulin isotype responsible for cytoskeleton organization. The authors showed that TUBB6 is essential in regulating microtubule and actin cytoskeleton dynamics in osteoclasts. Furthermore, this study developed a proteomic approach and identified proteins whose association with microtubules was affected by TUBB6.

Osteoclast activity and differentiation are controlled by their interaction with the osteoblastic lineage, a process termed coupling. Borggaard et al. investigated the spatial organization of several putative secreted and membrane-bound coupling factors and their receptors in human cortical bone remodeling processes. They identified coupling factors such as LIF, SEMA4D and PDGFB in human bone-resorbing osteoclasts and their respective receptors LIFR, PLXNB1, PDGFRA, and PDGFRB in neighboring reversal cells and osteoblasts. These findings support previous functional studies and highlight the critical role of these coupling factors in the osteoclast-osteoblast coupling in human bone remodeling. In their review, Russo et al. introduces the osteoclastogenesis process illustrating the reciprocal regulation of osteoclasts and other bone resident cells. To date multiple osteoclast precursor subsets and initiation of differentiation sites have been identified, contributing to osteoclast diversity.

### Perspectives in pharmacological targeting of osteoclasts for diagnosis and treatment of bone pathologies

The crosstalk of osteoclasts with other cell types not only contributes to bone homeostasis maintenance but can also exacerbate several pathological conditions leading to bone destruction. This is particularly the case in many chronic inflammatory diseases and in cancer.

As an example, Tao et al. emphasize the common pathways shared in diabetes and periodontitis by exploring the effect of the antidiabetic drug metformin on osteoblast and osteoclast formation and function *in vitro*. While metformin does not affect periodontal ligament-driven osteoblastogenesis, it effectively inhibits osteoclast formation by acting on the production of RANKL and M-CSF by fibroblasts and altering the differentiation of potential osteoclast progenitors. The link between chronic diseases and osteoclastogenesis was also demonstrated by Pramusita et al. in a murine model of salt-sensitive hypertension. This model is characterized by increased osteoclast formation and bone loss associated to a high TNF-α production and angiotensin II type 1 receptor upregulation in osteoblasts, which may contribute to increased RANKL levels. Another example of the importance of the interplay between osteoclasts and neighboring cells is provided by Lin et al. who explored the protective effect of the anti-inflammatory flavonoid acacetin in the context of osteoporosis. They showed that acacetin decreases osteoclast formation by interfering with RANK signaling pathway and stimulates the capacity of bone-marrow derived macrophages to promote angiogenesis, both *in vivo* and *in vitro*, limiting bone loss in osteoporotic mice. Ruocco et al. provide detailed information on the role of the complement C5a-C5aR1 axis in bone pathogenesis of the most common inflammatory bone diseases such as rheumatoid arthritis, as well as in osteoporosis and cancer. The authors discuss available C5aR1-based therapeutic approaches along with the importance of the C5a-C5aR1 axis in the interaction between the skeletal and immune system and its potential use as a new therapeutic target for the treatment of inflammatory bone diseases.

In their review, Russo et al. focused on osteoclast involvement in skeletal tumor progression. Osteoclasts can increase the aggressiveness of primary bone tumors or reinforce the bone metastatic process and reactivate dormant cancer cells in bone marrow niches. The authors discussed current therapies targeting bone resorption and osteoclastogenesis. Excessive bone turnover is typical of Paget’s disease and is associated with an increase in size and number of resorbing osteoclasts (Paget, 1877). Gennari et al. realized a comprehensive review on recent advances related to the pathogenesis of this disease. Although the mechanisms underlying the development of this disorder remain largely unknown, concomitant factors other than genetic susceptibility, such as viral infections and/or environmental factors, have been suggested as possible etiologies. The efforts toward a deeper understanding of this disease are driven by the lack of a resolutive cure, with current treatment being bisphosphonates (Lipton, 2005) and symptomatic medications for pain relief.

### Development in technology

In recent decades, significant advances have been made in the characterization of osteoclast physiopathology despite technology limitations. Even today, evaluation of osteoclast number, size and activity is greatly time consuming and exhibits operator-related bias despite having trained personnel, making continuous monitoring of osteoclast function difficult. Harnessing the potential of electric cell-substrate impedance sensing (ECIS), Jansen et al. developed an impedimetric bioassay for real-time resorption quantification. This allowed the monitoring of IL-1β-induced resorptive activity during osteoclast culturing, highlighting how this inflammatory factor accelerates resorption in the initial phase of stimulation, although final total resorption (generally quantified by classical methods) was unaffected. In addition, Kohtala et al. developed an automated method for the quantification of osteoclast number and dimension using machine learning-based object detection. All steps are executed by a specific software that performs single-cell analysis after pixel translation. The application of machine learning can partially overcome the user’s subjectivity in osteoclast evaluation. These highlighted contributions are only examples of the collective efforts in the direction of the standardization of reliable methods to quantify osteoclast number, size, and biological activities over time, not only *in vitro*, but also using non-invasive *in-vivo* approaches.

## Concluding remarks

This Research Topic prospects the central role of the osteoclasts in bone pathophysiology, collecting recent evidence from in-depth analysis of the mechanisms regulating osteoclast formation and activity, as well as their communication with the surrounding system. It provides a better understanding of the role of osteoclasts in chronic diseases and cancer associated with bone loss. In addition, this Research Topic confirms that osteoclast research continues to provide new data and concepts, as well as new perspectives, both at the fundamental level and in terms of new therapeutic opportunities.
